# Effect of comprehensive rehabilitation on pressure pain threshold, functional disability, and plantar pressure among type 2 diabetes mellitus individuals with chronic low back pain

**DOI:** 10.1177/10538127251387831

**Published:** 2025-10-21

**Authors:** Shetty Shrija Jaya, Shyamasunder Bhat N, Rajagopal Kadavigere, Belehalli Pavan, Ashu Rastogi, B Ananthakrishna Shastry, Shreemathi S Mayya, G Arun Maiya

**Affiliations:** 1 Centre for Podiatry & Diabetic Foot Care and Research, Department of Physiotherapy, Manipal College of Health Professions, Manipal Academy of Higher Education, Manipal, India; 2 Department of Orthopaedics, Kasturba Medical College - Manipal, Manipal Academy of Higher Education, Manipal, India; 3 Department of Radiodiagnosis and Imaging, Kasturba Medical College - Manipal, Manipal Academy of Higher Education, Manipal, India; 4 Department of Orthopaedics and Department of Podiatry, Karnataka Institute of Endocrinology and Research, Bangalore, India; 5 Department of Endocrinology and Metabolism, PGIMER, Chandigarh, India; 6 Department of Medicine, Kasturba Medical College - Manipal, Manipal Academy of Higher Education, Manipal, India; 7 Department of Data Science, Prasanna School of Public Health, Manipal Academy of Higher Education, Manipal, India

**Keywords:** chronic low back pain, photobiomodulation, plantar pressure, rehabilitation, type 2 diabetes mellitus

## Abstract

**Background:**

Chronic low back pain(CLBP) is the most prevalent musculoskeletal problem reported by individuals with Type 2 Diabetes Mellitus(T2DM). The effect of therapeutic intervention in this concomitant group of conditions has received limited scholarly attention.

**Objective:**

To evaluate the effect of a comprehensive rehabilitation on pressure pain threshold(PPT), functional disability, and plantar pressure in T2DM individuals with CLBP.

**Methods:**

In this study, 40 T2DM individuals with CLBP were included based on the pre-decided eligibility criteria. These participants received a comprehensive rehabilitation consisting of photobiomodulation, pain education, and exercise interventions for 12 weeks. The intervention's effect was evaluated on PPT, disability, and plantar pressure, which were assessed at baseline, the 12^th^ week, and the 24^th^ week. PPT was evaluated using an algometer. Oswestry disability index(ODI) and Win-Track system were used to assess disability and plantar pressure parameters, respectively.

**Results:**

A statistically significant improvement over the time points was noted in PPT (F_(1.72,67.07)_ = 117.9), ODI (F_(1.49,58.19)_ = 119), maximal plantar pressure (F_(1.62,63.09)_ = 33.01), and average plantar pressure (F_(1.41,55.11)_ = 19.87). No significant difference was observed in contact area of bilateral feet across different timepoints.

**Conclusion:**

A 12-week comprehensive rehabilitation program was effective in improving PPT, functional disability, and plantar pressure in T2DM individuals with CLBP.

## Introduction

According to the International Diabetes Federation (IDF) Atlas 2025 report, Type 2 Diabetes Mellitus (T2DM) has a significant worldwide prevalence, affecting approximately 588.7 million people in 2024, with projections estimating 852.5 million people by 2050.^1^ T2DM individuals suffer from a wide range of musculoskeletal conditions. Among these, chronic low back pain (CLBP) is the most common, with a reported prevalence of 19.8% to 34.8%.^2,3^ Chronic hyperglycemia resulting from longer uncontrolled T2DM leads to the accumulation of advanced glycation end products (AGEs) in the collagen of intervertebral discs, microvascular damage, and a state of chronic low-grade inflammation.^4–6^ Thus, the severity of CLBP is influenced by the duration of T2DM and level of glycemic control, with prolonged disease and poor regulation being associated with severe disc degeneration.^7^

The altered blood circulation seen in T2DM contributes to the connective tissue degeneration that increases the chances of CLBP and disc prolapse.^6^ Hyperglycemia in T2DM leads to the accumulation of AGEs, modifying extracellular matrix and cellular homeostasis and thus accelerating the aging of the disc. Additionally, AGEs are also responsible for a sustained inflammatory cascade that exacerbates tissue damage and pain.^4,5^ Hence, T2DM individuals with CLBP present with increased severity of pain, higher disability level, and poor health quality compared to non-T2DM individuals with CLBP.^8,9^ The increased pain and disability related to CLBP can alter the gait pattern and distribution of pressure across the foot.^10^ Plantar pressure is a critical factor in T2DM individuals; any abnormal distribution or increased plantar pressure can result in serious foot-related complications.^11^

Considering pain, disability, and foot complications in T2DM individuals, an appropriate rehabilitation targeting these factors becomes essential to manage the severity and its complications. Photobiomodulation is a non-invasive, safe light-based pain-relieving treatment commonly used for chronic musculoskeletal conditions.^12^ It improves cell proliferation, accelerates the healing process, and prevents cell death.^13,14^ The analgesic effect of photobiomodulation is purported to enhance microcirculation and modulate prostaglandin E2 levels, which play a vital role in the inflammation process.^15,16^ Sustained low-grade inflammation is a feature of T2DM, also responsible for chronic pain.^17^ However, there is limited scholarly attention on photobiomodulation as a treatment for CLBP in T2DM individuals. Pain education, along with exercise interventions, offers a comprehensive approach to managing CLBP, leading to effective and sustained improvement in pain and disability.^18^ Idowu et al. found that graded activity and monitored walking were effective in improving pain severity, disability, and glycemic control in T2DM individuals with CLBP for up to 12 weeks.^19,20^ Comprehensive rehabilitation for T2DM individuals is crucial due to the complex nature of the disease and its complications. Although there are studies that have evaluated the effect of therapeutic interventions in managing CLBP in T2DM patients, there is a dearth of literature on the effect of comprehensive treatment comprising photobiomodulation, pain education, and exercise interventions in this group. Hence, the purpose of this study was to evaluate the effect of a comprehensive rehabilitation on pressure pain threshold (PPT), functional disability, and plantar pressure in T2DM individuals with CLBP.

## Methods

### Study design

It was a single-arm intervention study. Approval for this study was obtained from the institutional research and ethics committee (IEC1306/2022), and the trial was registered under the Clinical Trial Registry of India (CTRI/2023/03/050346). This study was subsequently carried out from March 2023 to February 2024.

### Participants

This study was conducted in a tertiary care hospital located in the southern part of India. 199 T2DM individuals were screened, and 40 T2DM individuals with CLBP were recruited in this study based on the inclusion and exclusion criteria. The minimum requirement for the inclusion of participants in this study were as follows: age between 18 and 65 years, individuals who had been diagnosed with T2DM for ≥1 year prior to study enrollment and currently on medication, an HbA1c level of more than 6.5% (48 mmol/mol), having low back pain (LBP) for more than 3 months, and an oswestry disability index (ODI) score of more than 20%.^21^ Individuals with any infections or malignancy, systemic disorders, unstable cardiovascular or pulmonary conditions, any neurological or severe psychiatric disorder, pregnancy, acute disc prolapse, rheumatological conditions, scoliosis, vertebral fracture, cauda equina, or traumatic LBP were excluded. All the participants received a detailed explanation about the study procedure. All participants who were eligible and willing to participate provided written consent.

**Figure 1.**
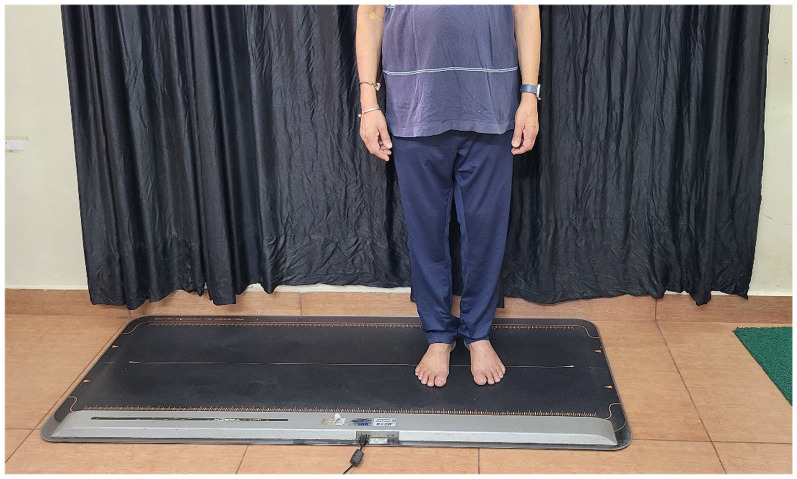
Assessment of plantar pressure using Win-Track system.

**Figure 2.**
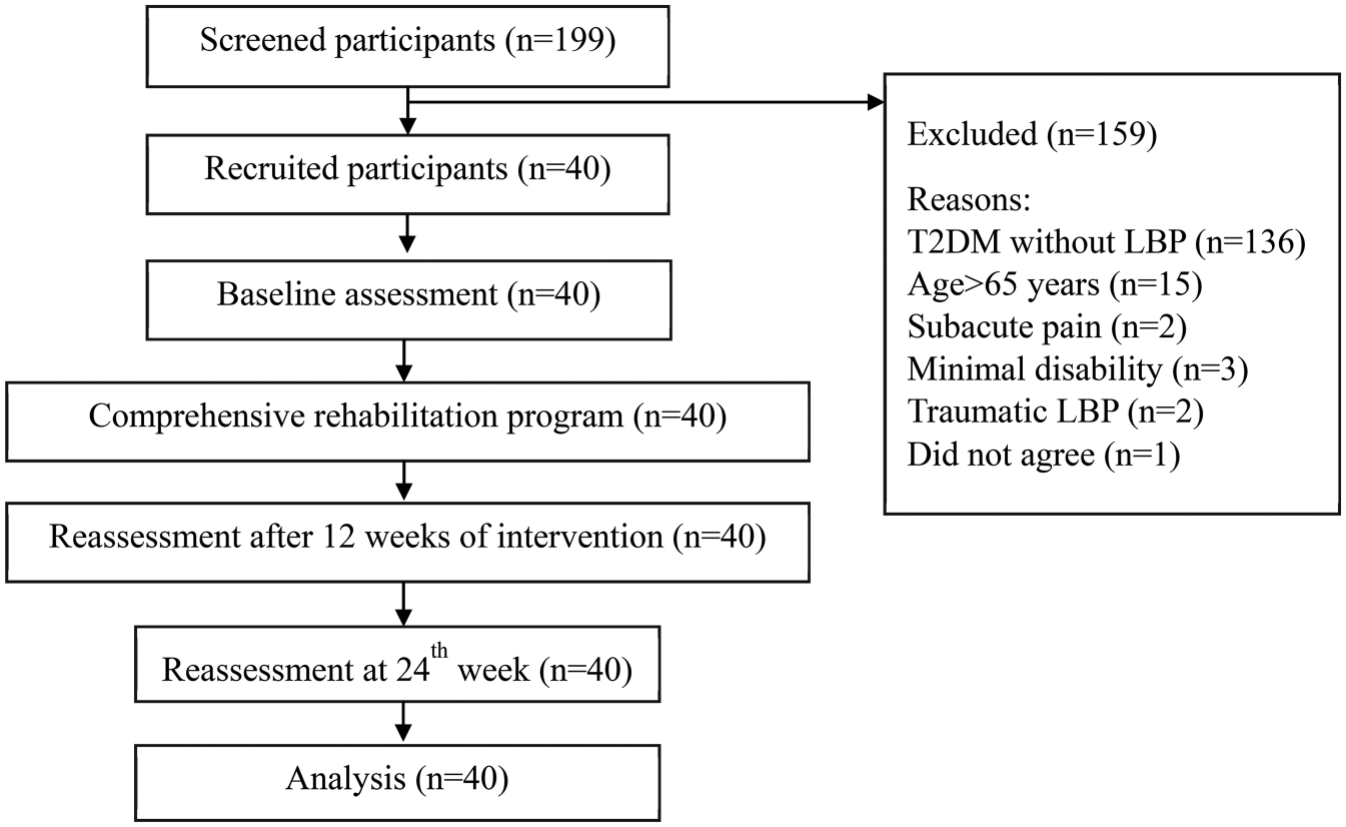
Flow diagram of the recruitment of participants.

### Intervention

All recruited 40 participants were provided with a comprehensive rehabilitation program for 12 weeks, which included photobiomodulation, pain education, and exercise intervention, including walking. From the 12^th^ to the 24^th^ week, participants were on the maintenance phase, during which they were asked to continue the same exercises without supervision. Photobiomodulation was given to the painful lumbar intradiscal or paraspinal area for 5–7 days to manage pain. Photobiomodulation treatment was provided using the THOR LX2 device with a combination wavelength of 660 and 905 nm, pulsed at 156 Hz for 3 min at each painful region.^22^ It was followed by two sessions of supervised pain education that included pain neuroscience education, modification of negative beliefs, and back care. Each session of pain education lasted for 30 min and was provided during the 1^st^ week of the treatment. Exercise interventions included trunk, proximal lower limb, and foot exercises. Trunk exercises included flexion and extension exercises, core activation and strengthening; proximal lower limb exercises targeted hip flexors, abductors, extensors, and rotators; and foot exercises included intrinsic muscles strengthening. These exercises were for 12 weeks and progressed as per type of exercise, resistance, and repetition at the 4^th^ and 8^th^ weeks. Exercises were supervised for the first week, followed by one supervised session once every two weeks. Each exercise was performed 5–10 repetitions per session. Participants were instructed to perform these exercises 5–7 times per week. Stretching of the erector spinae muscle, piriformis, rectus femoris, hamstring, and achilles tendon was included in this exercise intervention as a warm-up and cool-down exercise. Walking progressed from pain-free walking to up to 60 minutes per day. Regular telephonic calls were used to check exercise adherence.

### Outcomes

Baseline demographics and clinical data included age, duration of T2DM, and duration of LBP. Height, weight, blood pressure, and HbA1c level of participants were measured. The primary outcomes were PPT, functional disability, maximal plantar pressure, average plantar pressure, and contact area of the foot. These outcomes were measured at baseline, at the 12^th^ week (after completing 12 weeks of the rehabilitation program), and at the 24^th^ week (follow-up).

#### PPT

PPT was assessed using an algometer, which can measure the sensitivity of muscular tissue. An algometer is a valid instrument to measure pain sensitivity.^23^ The algometer used in this study was the ECHO algometer from JTECH Medical. The participant was instructed to lie on the plinth in a prone position. PPT was evaluated using a 1.0 cm^2^ algometer tip. The algometer's tip was positioned perpendicular to the skin on the paraspinal muscle at the L5 level of the spine, as it is a common site for biomechanical stress, increased degeneration, and soft tissue issues that contribute to CLBP.^24–26^ It was also observed as a common site of pain for most of the participants included. Pressure was gradually increased, and the subject was instructed to notify when the pressure was sensed as pain. The pressure at which the participant perceived it as pain was noted in Kg/cm^2^.

#### Functional disability

Functional disability was assessed using the Oswestry disability questionnaire. This questionnaire has 10 domains related to various activities. Each domain of activity has a rating from 0 to 5. Participants had to select each score based on their difficulty level performing each activity. It has a total score of 50 or 45, with one optional domain. ODI is the percentage of an obtained score. It is an acceptable tool to measure disability among LBP participants. ODI is a widely accepted, valid, and reliable tool to measure the effect of CLBP on an individual's function and quality of life.

#### Plantar pressure

Plantar pressure was assessed using the Win-Track device from Medicapteurs, France. This system has good reliability in assessing plantar pressure parameters.^27^ The Win-Track system has a pressure platform with 12,288 sensors and software. Participants were positioned barefoot on the platform with their arms relaxed by their sides (Figure 1). Pressure parameters such as contact area, maximal pressure, and average pressure were obtained and analyzed using Win-Track software. The unit of contact area was cm^2^, and pressure was kilopascal (kPa).

### Sample size

The sample size was calculated using online G*Power 3.1.9.7. With an alpha level (α) set at 0.05, an effect size of 0.3, a power of 90%, one group, and three measurement timepoints, the calculated sample size was 35. Therefore, a total of 40 participants were recruited for this study.^28^

### Data collection process

Demographic, clinical, and baseline assessments of the participants were conducted on the first day of recruitment to the study after receiving the signed informed consent. The outcome assessments for all participants were performed by an experienced physiotherapist. These evaluations were carried out on the first day, at the end of the 12^th^ week, and 24^th^ week by the same therapist.

### Statistical method

Data was analyzed using Jamovi 2.3.21 software. Descriptive statistical tests were used to summarize demographic and clinical data. The normality of data was assessed using the Shapiro-Wilk test. If data followed normal distribution, then repeated measures ANOVA was used to compare the data or evaluate the effect of intervention at three different timepoints. For sphericity correction, Greenhouse-Geisser was applied for all the primary outcomes. The Tukey post hoc analysis was used to find the significant difference between two specific time points. The significance level was set at p < 0.05.

## Result

After screening 199 participants, 40 participants were recruited for this study (Figure 2). The mean age of the 40 included participants was 59.6 years. There were 22 females (55%) and 18 male participants (45%). The mean duration of T2DM was 9.66 years. Table 1 presents the clinical characteristics of the participants.

**Table 1.** Clinical characteristics of the participants.

Characteristics (n = 40)	Mean ± SD
Age (years)	59.60 ± 4.46
BMI (Kg/m^2^)	26.43 ± 4.14
Systolic blood pressure (mmHg)	136.38 ± 12.83
Diastolic blood pressure (mmHg)	82.38 ± 8.10
Duration of T2DM (Years)	9.66 ± 7.45
HbA1c (%)	8.05 ± 0.95
Duration of LBP (months)	25.38 ± 34.85

SD- Standard deviation, BMI- Body mass index, T2DM- Type 2 Diabetes Mellitus, HbA1c- Glycated Haemoglobin, LBP- low back pain

### Effect of comprehensive rehabilitation on PPT

**Figure 3.**
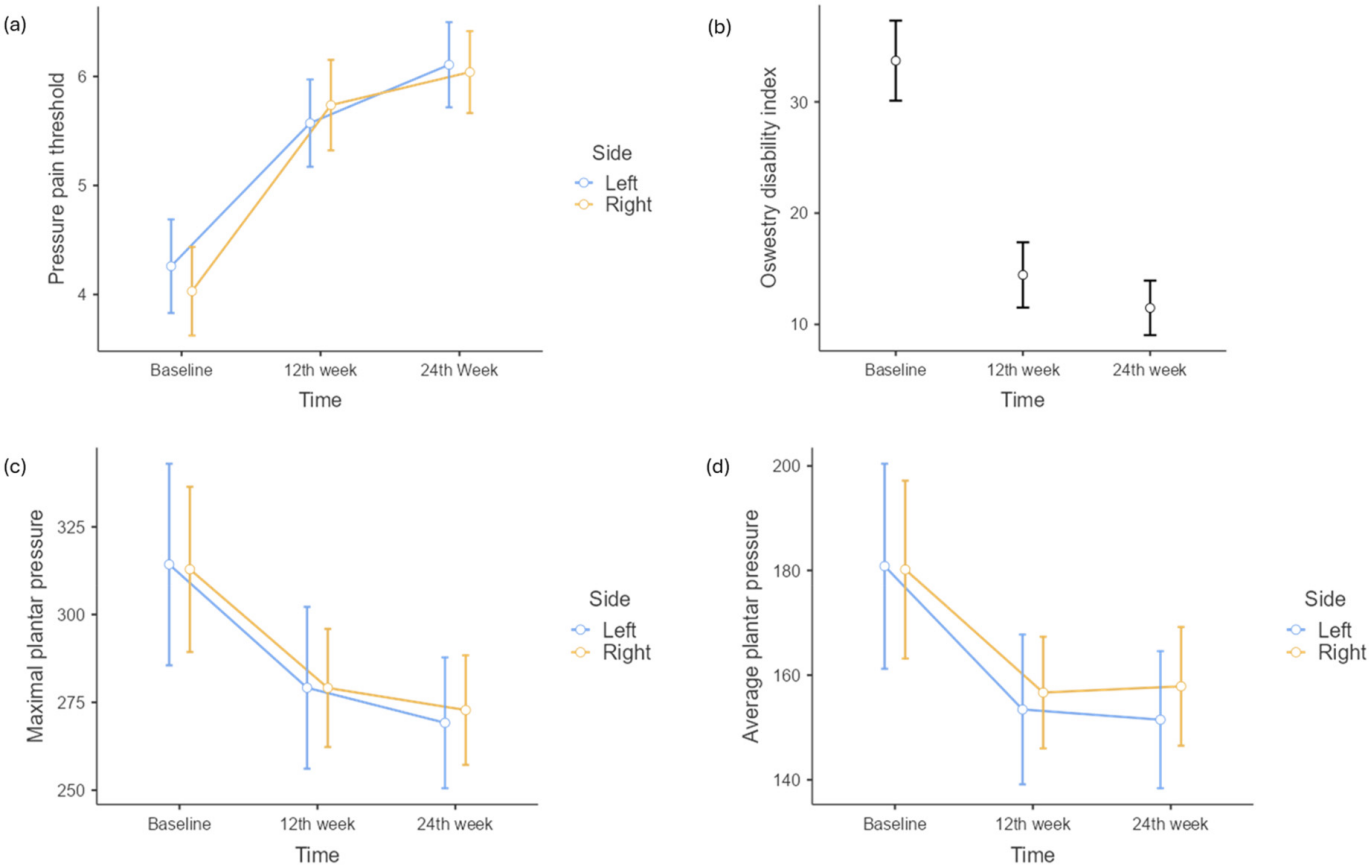
Graph of change in means of PPT, ODI, maximal and average plantar pressure at three timepoints.

Repeated measures ANOVA showed a statistically significant difference in means of PPT with respect to different timepoints [F_(1.72,67.07)_ = 117.9, p < 0.001, η^2^p = 0.75] (Table 2). Tukey's post hoc test revealed a significant increase in mean PPT at the 12th week from baseline (t = 10.38, p_tukey_ < 0.001) and at the 24^th^ week from baseline (t = 13.38, p_tukey_ < 0.001) (Figure 3).

**Table 2.** Effect of comprehensive rehabilitation on PPT, disability and plantar pressure.

Outcomes		Baseline	12^th^ week	24^th^ week	p-value
PPT	Left side at L5	4.26 ± 1.34	5.57 ± 1.25	6.11 ± 1.22	<.001
	Right side at L5	4.03 ± 1.27	5.74 ± 1.30	6.04 ± 1.18	<.001
Functional disability	ODI	33.7 ± 11.25	14.4 ± 9.17	11.5 ± 7.66	<.001
Plantar pressure	Left area	20.8 ± 5.69	21.8 ± 5.93	20.6 ± 4.02	0.293
	Left maximal pressure	314.3 ± 89.85	279.1 ± 72.01	269.2 ± 58.22	<.001
	Left average pressure	180.8 ± 61.31	153.4 ± 44.71	151.5 ± 40.91	<.001
	Right area	20.0 ± 5.78	20.4 ± 4.23	20.0 ± 4.00	0.816
	Right maximal pressure	312.9 ± 73.60	279.1 ± 52.58	272.8 ± 48.75	<.001
	Right average pressure	180.2 ± 53.18	156.7 ± 33.31	157.9 ± 35.46	<.001

PPT- Pressure pain threshold, ODI- Oswestry disability index.

### Effect of comprehensive rehabilitation on functional disability

A statistically significant difference in means of ODI at various timepoints was noted using repeated measures ANOVA [F_(1.49,58.19)_ = 119, p < 0.001, η^2^p = 0.75] (Table 2). Tukey's post hoc test showed a significant decrease in mean ODI at the 12th week compared to baseline (t = 11.79, p_tukey_ < 0.001) and at the 24^th^ week from baseline (t = 11.80, p_tukey_ < 0.001). A significant minimal difference was noted between the 12^th^ and 24^th^ week (t = 2.81, p_tukey_ = 0.02) (Figure 3).

### Effect of comprehensive rehabilitation on plantar pressure parameters

Repeated measures ANOVA indicated a significant difference in means of maximal plantar pressure at different timepoints [F_(1.62,63.09)_ = 33.01, p < 0.001, η^2^p = 0.45] (Table 2). In addition, post hoc analysis using Tukey's test showed a significant reduction in mean maximal plantar pressure at the 12th week compared to baseline (t = 6.30, p_tukey_ < 0.001) and at the 24^th^ week from baseline (t = 6.38, p_tukey_ < 0.001). However, there was no significant difference between the means of maximal plantar pressure at 12^th^ and 24^th^ week (t = 1.89, p_tukey_ = 0.15) (Figure 3).

Repeated measures ANOVA showed a significant change in means of average plantar pressure across various timepoints [F_(1.41,55.11)_ = 19.87, p < 0.001, η^2^p = 0.33] (Table 2). Tukey's post hoc analysis revealed a significant reduction in means of average plantar pressure at the 12th week from baseline (t = 4.74, p_tukey_ < 0.001) and at the 24^th^ week from baseline (t = 4.75, p_tukey_ < 0.001). However, no significant difference was observed between the average plantar pressure at 12^th^ and 24^th^ week (t = 0.136, p_tukey_ = 0.99) (Figure 3). Repeated measures ANOVA did not reveal any statistically significant difference in means of contact area at any timepoints [F_(1.47,57.28)_ = 0.93, p = 0.372, η^2^p = 0.02] (Table 2).

## Discussion

In this pre-post study, we evaluated the effect of a comprehensive rehabilitation program on PPT, functional disability, and plantar pressure parameters among 40 T2DM individuals with CLBP. Participants received comprehensive rehabilitation consisting of photobiomodulation therapy, pain education, and exercise interventions targeting trunk, lower limb muscles, and walking for 12 weeks.

### Effect of comprehensive rehabilitation on PPT

T2DM individuals with CLBP experience severe pain symptoms and disability compared to the general population. The presence of CLBP affects their regular physical activity and exercise participation, which is crucial to managing diabetes. The pain, therefore, impacts the self-care management required for glycemic control in T2DM individuals.^29^ PPT is a measure to detect the minimal pressure at which an individual perceives pain.^30^ Individuals with chronic pain conditions such as CLBP exhibit lower PPT values compared to healthy individuals. They often present with hyperalgesia, indicating higher sensitivity to pain.^31^ Similarly, T2DM is a chronic condition in which patients usually suffer from neuropathic pain that can lower PPT values.^32^ Improving PPT can indicate reduced pain sensitivity and better pain management, which is important for T2DM individuals with CLBP to manage self-care routines.^33^ In our study, a statistically and clinically significant increase in PPT value was noted at the 12th week (MD = 1.51) and 24th week (MD = 1.92) from baseline following a comprehensive rehabilitation program.^33^ The increase in PPT values can be attributed to the combined effect of photobiomodulation and exercise intervention, which have modulated the inflammatory process, improved ATP synthesis, and enhanced tissue repair, healing, and recovery of spinal tissues and muscles, leading to a reduction in pain sensitivity.^34,35^ Higher PPT values are associated with improved function and reduced disability in individuals with chronic pain.^33^

### Effect of comprehensive rehabilitation on functional disability

Individuals with T2DM present poorer patient-reported outcomes compared to those without T2DM, indicating the negative effect of T2DM on disability in CLBP individuals.^36^ Increased functional disability in T2DM individuals with CLBP can significantly impact their everyday activities by limiting their physical abilities, increasing psychological stress, and reducing overall quality of life. In our study, a significant reduction in the mean ODI value by 19.26% was noted at the 12^th^ week of assessment, and a mean difference of 22.22% was reported from baseline to the 24^th^ week. These differences were more than the established MCID value of 11%, suggesting the favourable outcome of the provided comprehensive rehabilitation.^37^ The effect of the rehabilitation was maintained from the 12^th^ to the 24^th^ week of assessment, with a minimal significant change of 2.97% ODI. A previous study by Shi et al. indicated that T2DM individuals with degenerative lumbar spinal stenosis showed a significant improvement in low back disability following therapeutic exercises for 6 weeks, but no difference in the ODI improvement was seen when compared with the non-T2DM group.^28^ In line with our study, a study by Idowu et al. demonstrated a significant improvement in LBP-related disability at 8 weeks with 12 weeks of graded activity and daily monitored walking in T2DM individuals with persistent LBP.^19^ A combined effect of pain management, including pain education and exercise intervention targeting core and lower limb muscles, might contribute towards functional recovery and mobility, thereby decreasing the LBP-related disability in T2DM individuals.

### Effect of comprehensive rehabilitation on plantar pressure

LBP causes abnormal spine proprioception, altered activation of muscle, and reduced neuromuscular control.^38^ The abnormal performance of the lumbopelvic hip complex, core muscle insufficiency, altered posture, and gait due to CLBP can lead to abnormal plantar pressure distribution.^39^ Plantar pressure is a critical factor in managing foot health in T2DM individuals. Increased plantar pressure is a risk factor for the development of foot ulcers, leading to severe complications such as infections and amputations. CLBP, along with diabetic neuropathy, can significantly alter plantar pressure in T2DM individuals as they lack protective sensations that help in identifying increased plantar pressure or any injury, potentially exacerbating the risk of foot complications.^11^ Our study found a significant reduction in maximal and average plantar pressure at 12^th^ and 24^th^ weeks from baseline following a comprehensive rehabilitation program (p < 0.001). The reduction in the maximum and average plantar pressure can be related to the normal recruitment of core and lower limb muscles with the 12 weeks of exercise intervention. This might have led to an even distribution of plantar pressure and a reduction in the peak pressure at certain points in the foot. No significant change was noted from the 12^th^ to the 24^th^ week in these parameters. These insignificant changes could be due to the discontinuation of exercises post 12 weeks of rehabilitation. However, the effect of rehabilitation lasted 24 weeks without an increase in the plantar pressure parameters. There was no change reported in the contact area of the foot following comprehensive rehabilitation. This result could be due to the redistribution of pressure across the foot without much change in the total surface area in contact with the platform. Overall, there was a positive effect of the rehabilitation on the plantar pressure parameters.

In this study, the use of analgesics during the intervention was recognized as a potential confounding factor that may have an influence on pain perception and outcomes. This has been identified as one of the limitations of our study. Adherence to the treatment was checked regularly through phone calls and the documentation of the exercise performed by each participant. Participants were motivated and encouraged regularly at intervals to follow the recommended exercises, making sure that there was no deviation from the treatment plan. Overall, comprehensive rehabilitation has provided beneficial effects to the participants. The implementation of this rehabilitation program will help reduce pain and disability associated with CLBP in T2DM individuals. This will allow them to engage more actively in daily activities, promoting functional recovery and improving diabetes management.

### Limitations and future recommendations

This study had a few limitations. It did not consider the effect of analgesics or diabetes medications, and the diet followed by the participants. The attrition rate was not considered in the sample size calculation. It did not have a long-term follow-up to evaluate the recurrence of pain and maintenance of function. This study lacked a control group. Hence, future studies can include a control group to compare the extent of effectiveness. Studies can also include a radiographic investigation to study the effect of rehabilitation at the spinal level.

## Conclusion

A comprehensive rehabilitation consisting of Photobiomodulation therapy, pain education, and exercise intervention, including walking, is effective in improving PPT, functional disability, and plantar pressure distribution among T2DM individuals with CLBP. The higher prevalence and increased disability related to CLBP among T2DM individuals make it crucial to implement this rehabilitation at an early stage to prevent further complications and poor function.
